# Uptake and Accumulation of Nano/Microplastics in Plants: A Critical Review

**DOI:** 10.3390/nano11112935

**Published:** 2021-11-02

**Authors:** Imran Azeem, Muhammad Adeel, Muhammad Arslan Ahmad, Noman Shakoor, Gama Dingba Jiangcuo, Kamran Azeem, Muhammad Ishfaq, Awais Shakoor, Muhammad Ayaz, Ming Xu, Yukui Rui

**Affiliations:** 1 Beijing Key Laboratory of Farmland Soil Pollution Prevention and Remediation, College of Resources and Environmental Sciences, China Agricultural University, Beijing 100193, China; imranazeem19@gmail.com (I.A.); lb20203030039@cau.edu.cn (N.S.); 2 BNU-HKUST Laboratory of Green Innovation, Advanced Institute of Natural Sciences, Beijing Normal University Zhuhai Subcampus, Zhuhai 519087, China; karma010@163.com; 3 Shenzhen Key Laboratory of Marine Bioresource and Eco-Environmental Science, College of Life Sciences and Oceanography, Shenzhen University, Shenzhen 518060, China; arslan.slu@gmail.com; 4 Department of Agronomy, the University of Agriculture Peshawar, Peshawar 25000, Pakistan; azeemmkd@gmail.com; 5 College of Resources and Environmental Sciences, National Academy of Agriculture Green Development, Key Laboratory of Plant-Soil Interactions, Ministry of Education, China Agricultural University, Beijing 100193, China; ishfaq@cau.edu.cn; 6 Department of Environment and Soil Sciences, University of Lleida, Avinguda Alcalde Rovira Roure 191, 25198 Lleida, Spain; awais.shakoor@udl.cat; 7 Lithuanian Research Center for Agriculture and Forestry Instituto al. 1, 58344 Akademija, Lithuania; muhammad.ayaz@lammc.lt

**Keywords:** bioavailability, nanoplastics, microbes, rhizosphere, translocation

## Abstract

The ubiquitous presence of microplastics (MPs) and nanoplastics (NPs) in the environment is an undeniable and serious concern due to their higher persistence and extensive use in agricultural production. This review highlights the sources and fate of MPs and NPs in soil and their uptake, translocation, and physiological effects in the plant system. We provide the current snapshot of the latest reported studies with the majority of literature spanning the last five years. We draw attention to the potential risk of MPs and NPs in modern agriculture and their effects on plant growth and development. We also highlight their uptake and transport pathways in roots and leaves via different exposure methods in plants. Conclusively, agricultural practices, climate changes (wet weather and heavy rainfall), and soil organisms play a major role in transporting MPs and NPs in soil. NPs are more prone to enter plant cell walls as compared to MPs. Furthermore, transpiration pull is the dominant factor in the plant uptake and translocation of plastic particles. MPs have negligible negative effects on plant physiological and biochemical indicators. Overall, there is a dire need to establish long-term studies for a better understanding of their fate and associated risks mechanisms in realistic environment scenarios for safe agricultural functions.

## 1. Introduction

**Figure 1:**
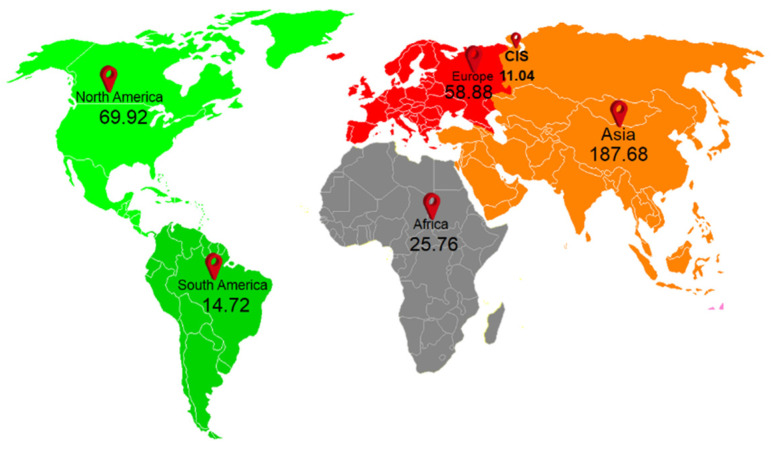
Worldwide production of plastics, numbers indicate in million tons. (The data is obtained from [6]).

Plastics are synthetic materials made up of polymers, which are long molecules around chains of carbons atoms, especially hydrogen, nitrogen, oxygen, and sulfur [1,2]. Plastics can be categorized based on their size, i.e., microplastics (>25 mm), mesoplastics (5–25 mm), microplastics (MPs) (0.1–5 mm), and nanoplastics (NPs) (<100 nm) [3]. Overall, worldwide plastic production is approximately 6300 million tons, of which 79% is deposited in landfills and other ecological segments [4]. In 2019, global plastic production was 368 million tons; 114.08 million tons of plastic were produced only in China and 58.88 million tons only in Europe, as shown in Figure 1, and its capacity is expected to double in 2040 [4,5,6].

Plastic is part of our daily lives; approximately 4 trillion plastic bags are used annually and 1 million plastic bottles are consumed every single minute [7]. These plastic bags or bottles are used for ~12 min and need years to decompose [8]. Unauthorized dumping and inadequate waste management lead to the release of environmental plastics and have a long environmental lifespan, which can easily accumulate in different environmental matrices [9]. MP contamination is a rapidly increasing concern throughout the world and has been listed as the second most emerging environmental and ecological issue [10,11,12] after global warming [13].

**Figure 2:**
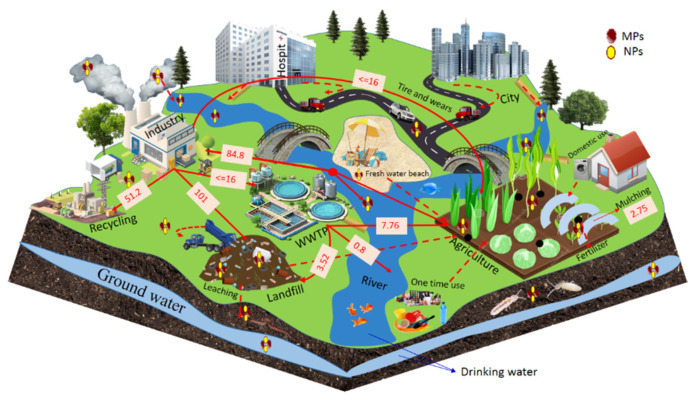
Estimates of plastic sources and transport pathways in the environment as reported in scientific literature; the data is indicated in million tons as well as the average data of high and low transportation in the ecosystem, arrows represent the known transportation and dashes represent unknown transportation in the ecosystem.

Soil, especially arable soil, has become a major and permanent sink for plastic, coming mostly from anthropogenic activities such as manufacturing {25.6–28% (Figure 2)} [4,14,15,16,17]. The extreme level of plastic pollution from wastewater treatment plants (WTPs) found on agricultural land is approximately 7.76 million tons [18]. In 2012, plastics being added to the soil through mulching by agricultural systems was equivalent to around 4.4 million tons [19,20]. Additionally, landfills sites also introduced plastics to the terrestrial ecosystem, but the exact data are not reported yet [21,22].

In territorial environments, MPs and NPs have varied toxic effects depending on the medium of exposure and interplay with other contaminants. Interactions with these pollutants can cause significant changes in the properties of plastic surfaces, and the agroecosystem can uptake these MPs and NPs and/or pollutants [23,24,25]. Co-exposure and the accumulation of these contaminants have been associated with antagonistic [26], synergistic [23,27,28], or additive effects [29]. Given the multifaceted nature of MPs and NPs, the study of contaminant interactions with MPs and NPs is a major concern in agroecosystem impact assessments. Raising chemicals, e.g., polybrominated diphenyl ether (PBDE), bisphenol A (BPA), and phthalates are commonly used to change the quality and performance of plastic. Nevertheless, plastics continue to deteriorate even after they are released into the ecosystem, and the use of these chemicals in synthetic plastic is more risky to animals, plants, and humans [30,31,32,33,34,35,36].

Agroecosystems are very prone to plastic contamination due to modern farming practices. Despite the potential entry of these emerging contaminants into the agricultural system, information on the impacts of MPs and NPs on soil biota, especially plants, is currently very scarce. As far as we know, only three studies have documented the effects of MPs and NPs on non-vascular plants [37,38,39] and only ten studies regarding the vascular plant have just been reported in the scientific literature [40,41,42,43,44,45,46,47,48,49]. Current literature detected the impact of MPs and NPs on bio-mass production and plant growth [49,50,51,52,53]. Detailed studies of NPs on wheat (*Triticum aestivum*) and broad beans (*Vicia faba*) have been published by Lian et al. [54] and Jiang et al. [48], respectively; moreover, two recent studies have investigated the effect of foliar application of NPs on the lettuce (*Lactuca sativa*) and maize (*Zea mays*) plants [46,47], which briefly discuss the toxic effects on plants.

We present a critical review of the previous 10 years; most of our cited literature spans the last five years of the effect of MPs and NPs on agroecosystems. Apparently, to the best of our knowledge, this is the first critical review on the uptake and accumulation of MPs and NPs in the plant system. First, we evaluate the abundance, sources, and distribution of MPs and NPs in agroecosystems. Secondly, we identify the fate and transport of MPs and NPs in the plant–soil system and their interaction with soil microorganisms. Thirdly, we examine the uptake and accumulation of MPs and NPs in plants (roots and shoots) and also their toxic impact on agricultural plants. In addition, this study could help environmental regulatory authorities improve their strategies and laws rereading MP and NP pollution in the agroecosystem.

## 2. Sources of Plastic in Agriculture Systems

Over the past six decades, plastics have become an essential and versatile product with a wide range of properties, chemical compositions, and applications. However, the release of plastic particles generated by the mass production of plastics is a major threat to the environment [55,56].

Plastic pollution, especially in the soil environment, adversely affects soil organisms and plants [57]. Plastic accumulates in the soil in different ways including through plastic packing, wastewater treatment plants, mulching, atmospheric deposition, and daily use products. The countless use of disposable plastic products cannot be separated from the serious pollution of MPs and NPs in soil [58].

In the market, the largest source of plastic comes from packaging [59] and with the rise of plastic resins, consumer marketplaces are getting more plastics day by day [60]. The use of plastic in packaging has rapidly increased and has made important contributions to facilitating daily life. Recent estimates show that more than 90 billion polyethylene bags are eliminated annually as non-renewable waste and trash [61]. The International Energy Agency (IEA) predicted that strong economic growth [62] will further increase the share of plastic packaging, i.e., 26% of the total volume in 2015, and could be doubled in the next 15 years with the increase of forecasted growth by up to four times by 2050 and up to 318 million tones/year, which is more than the entire current plastic industry [63]. Widespread use of plastics, known as “white pollution,” is becoming more serious in the environmental ecosystem [64]. All plastic that goes to landfills eventually reaches terrestrial/aquatic ecosystems.

The main sources of MPs and NPs in sewage wastewater treatments are fibers from clothing, microbeads from personal care products, cleaning products, and plastic debris [65,66,67,68,69]. A previous study determined that acrylic (17%) and polyester (67%) fibers were the main components of plastics in wastewater samples. Compared to actual waste material, it was found that the ratio was equivalent to the composition of textiles (5% acrylic and 78% polyester). Finally, it was concluded that sewage MPs come primarily from washing clothes [70]. In addition, through domestic washing machines, each wash can produce N1900 fibers. Usual wastewater treatment can release 300 million plastic debris into nearby watercourses every day [71]. Li et al. [72] showed that 90% of MPs accumulate in wastewater sludge and that its concentration in sludge ranges from 1500 to 56,400 particles kg^−1^ [73]. Most obviously, sludge is applied as fertilizer on agricultural land [69]. Similarly, another study documented that organic fertilizer has up to 895 particles kg^−1^ of MPs [74]. Hence, the continuous application of organic fertilizers and sludge can cause soil contamination with MPs [75,76], upsurge the contamination of extreme levels of farmlands, and are also harmful to plant and human health. The formation of a separate drainage system is of great importance for the immediate promotion of improved sludge treatment and handling services in agricultural systems.

In many countries, plastic mulching has become a widely adopted practice in agriculture for its immediate economic benefits [77]. On agricultural land, plastics, especially low-density polythene (LDPE), are used for mulching to enhance fruit and vegetable production. Undoubtedly, plastic mulching gives short-term benefits to growers by improving water-use efficiency in semi-arid regions, controlling some weeds, and earlier and late maturity of crops [78]. On the other hand, most of the world’s productive and valuable soil resources are exposed to plastic residue. For example, in China, about 20 million hectares of agricultural land are being exposed to mulching by plastics and is expected to increase by 7.1% or more per year [64,78]. In 2015, 1.455 million tons of plastic were used for mulching in China [79].

Once plastic accumulates in soil, it is technically very difficult to recycle or remove from the site because of its small size (0.01–0.03 mm). The residual films in the field could slowly break down into MPs and NPs with a combination of ultraviolet radiation and physio-chemical and biological possessions, resulting in MP and NP contamination in the soil [80,81].

Atmospheric deposition is another source of MPs and NPs entering surface soil. Atmospheric precipitation of plastic in the urban areas of Paris was around 2–35 particles m^−2^ days^−1^ [82]. Allen et al.’s [83] findings reported through air mass trajectory analysis that through atmospheric transportation, microplastic transport through the atmosphere at a distance of up to 95 km could reach and affect remote and sparsely populated areas. Microplastic detection in soil from remote/unsettled areas or restless high mountain areas has been reported [84].

Several studies documented that NPs are released from the degradation of plastic into the natural environment [85,86,87]. Studies by Ekvall et al. [88] revealed that MPs and NPs are produced during the mechanical degradation of daily use products. Lambert and Wagner [58] reported that MPs and NPs released in the degradation process of disposable cup lids constitute up to 1.26 × 10^8^ particle mL^−1^ over 56 days. Mechanical and photo-oxidative degradation leads to the release of MPs and NPs into the natural environment. Literature reported that UV-light irradiation degraded PS components (PS foam, single-use plates, and coffee cup caps) into the soil environment [58] and is harmful to plants and soil organisms. Plastics appear in agricultural soil through different sources; however, the exact concentration of MPs and NPs from different sources are variable and as the study of the source is at an early stage, the exact source for the soil in various land uses is still unclear.

## 3. The Fate of Plastic in Agricultural Soil

Plastic has been reported both in surface and subsurface soil [84,89,90,91,92]. Different agricultural practices including tillage, irrigation, as well as soil organisms promote plastic transport in different soil layers [92,93]. Deep tillage, moldboard, and deep plowing methods disturb different layers of soil and promote the deep penetration of MP and NP in subsoils [92,94,95]. In addition, the pruning of rhizomes such as potatoes and carrots can also support MPs and NPs to migrate downward [96]. A current study found that wet-dry circles can promote MPs to move downward. Dry climate induces soil cracks, which could facilitate the plastic to move in deep soil [97]. Zubris and Richards [75] found evidence that fibers move downward but transport mechanisms are still unknown.

Cey et al. [98] reported that the average diameter of MPs that can leach down into deep soil of up to 70 cm is 3.7 mm. In addition, the penetration of water flow, such as rain or irrigation water from top to bottom in the ground, transmits MPs and NPs to the bottom with the soil vacuum and eventually leads to the groundwater [93,99].

Soil organisms are the most representative factor to transport MPs and NPs in deep soil [100]. There is growing evidence that soil organisms such as earthworms (*Lumbricus terrestris*), mites (*Hypoaspis aculeifer*, *Damaeus exspinosus*), and collembolans (*Folsomia candida*) can help migrate MPs and NPs from topsoil to deep soil [92,101,102,103,104,105,106]. Soil organisms can carry MPs and NPs through their casting, burrowing or ingestion, egestion and pushing, and also adhesion to their exterior [107]. Rilling et al. [108] reported that earthworms (*Lambricus terrestris*) added 35 to 73% of MPs (<50 μm and 63–150 μm in diameter) from surface debris to their burrows and also transported much smaller particles (<50 μm) into deep soil [92]. In topsoil polyethylene, MPs were penetrated up to 10 cm while NPs (710–850 μm) were mostly observed in the deepest layer. This result shows that earthworms transport plastic in terms of size and NPs penetrate deeper in the soil as compared to MPs. Earthworms can also pull large and microscopic plastic particles down into their burrows without eating the pieces of plastic [76]. The vertical movement of soil organisms creates micropores in the soil, which promotes the transportation of MPs and NPs by leaching. Yu et al. [103] reported that MPs are moved up to 50 cm below due to the bioturbation of earthworms in sandy soil and further leaching encouraged the MPs to reactivate even the largest part (250–1000 μm) which was found in a 60 centimeter-high leachate column.

### 3.1. Bioavailability

MPs and NPs present in soil in different forms and [109] their bioavailability depends on soil properties such as particle size, particle density, abundance/co-occurrence, chemical characteristics, and the specific characteristics of the receptor (plant or organism) [110,111]. The bioavailability of MPs increases with its size, and a wide range of organisms directly ingest it [112]. As their size fraction is similar to that of sediments and planktonic organisms, planktivores can directly eat MPs during normal food behavior as natural prey [111]. Soil chemical properties such as pH, sorption and adsorption of heavy metals, and their redox potential perform a vital role in the bioavailability of plastic in soil [113,114,115]. Furthermore, iron–manganese oxide-bound fractions of nickel (Ni), copper (Cu), chromium (Cr), and exchangeable carbonated-bound fractions in soil can be reduced by the presence of MPs [114]. The presence of high-density polyethylene (HDPE), polystyrene (PS), polyethylene (PE), and polylactic acid (PLA) increases the diethylenetriamine pentaacetate (DTPA) extractable Cd concentration of soil [116,117,118]. The presence of plastic in soil alters the bioavailability of other metals; arsenic bioavailability was limited in the presence of MPs [119]. MPs serving as the vector of heavy metals were reported in terrestrial systems. Adsorption behaviors and underlying mechanisms of HMs by MPs are critical to understand potential risks [120]. Earthworms increase the bioavailability of Cd in MP-enriched soils [96]. Wang et al. [117] reported the higher bioavailability of Cd in the presence of PLA in soil–plant systems as compared to PE.

MPs and NPs contain harmful substances including pesticides, polybrominated diphenyl ethers (PBDEs), endocrine-disrupting chemicals (EDCs), polycyclic aromatic hydrocarbons (PAHs), phthalates, and bisphenol-A that are transported to soil systems and leach down to subsoil based on temperature, ultraviolet radiation, soil pH, oxygen content, and dissolved organic matter content [55,107,121,122]. Sorption of EDCs and other substances on the surface of MPs and NPs can disrupt decomposition [123]. EDC threshold toxicity is difficult to measure because of non-monotonic dose effects and low dose effects [124]. Large amounts of toxic chemicals are associated with recycled plastics compared with virgin plastic [121]. PS MPs adsorb dibutyl phthalate, which has negative effects on green microalgae [125]. Wastewater and bio-solids have strong sorption of toxic substances from MP and NP particles [126,127,128]. A large amount of non-polar hydrophobic substances such as PCBs reportedly adsorb aged pellets of MPs compared to fresh pellets [129]. Heavy metals, dichlorodiphenyltrichloroethane (DDT), PAHs, and persistent organic pollutants (POPs) are adsorbed within MP and NP particles. Chemicals adsorbed on the surface of MPs and NPs are more toxic as compared to those directly coming from plastics. For example, it can be seen that the sorption of PS MP on metals is in the order of Pb^2+^ > Cu^2+^ > Cd^2+^ > Ni^2+^ [130]. Under the same conditions, the rate of Cr released from MPs was found to be faster than the rate of lead (Pb) release [131]. This may be due to different hydration ionic radii and differences in divalent cation complexing ability [132]. The composition of MPs influencing the adsorption, diffusion, and release of heavy metals depends on their morphology, specific surface area, surface charge, and porosity [133,134,135]. Wang et al. [116] reported that PE MPs adsorbed five pesticides (diflubenzuron, difenoconazole, carbendazim, malathion, and dipterex) in agricultural soil through exothermic and spontaneous processes; these results show that PE MPs can transfer different types of pesticides in agricultural soils.

The pH value can change the zeta potential of MPs or heavy metal precipitation, thereby increasing or decreasing the adsorption of certain metals. Generally, pH value increases with the decreasing zeta potential of MPs. However, if the MP’s zero-charge point is below the pH of the water, the MP charge will be negative. Thus, the electrostatic attraction between the metals and the polymer increases. In contrast, precipitation of some metals may occur in environments with a pH > 7. A recent study reported that pH increases with increasing the adsorption of Cu, Zn, Ni, Cd, Pb, and cobalt (Co) by MPs [132,136,137,138,139,140,141,142]. These increases may be due to an increased charge on MPs. In contrast, the adsorption of Cr^+6^ by MP is found to decrease with an increase in pH. This may be due to the relatively weak Coulombic interaction between the Cr^+6^ oxyanion form and the MP with reduced surface positive charge [143].

Plastic particles carry charge themselves which can enhance their adsorption in plant roots due to electrostatic attraction, affecting nutrient immobilization or photosynthesis processes [54]. The adsorption of Cd into PS MPs and PS NPs is associated with the reduction of the negative charge that they carry. Lian et al. [45] reported greater Cd bioaccumulation in wheat seedlings because of low Cd concentration in a PS NPs–Cd solution. PE MPs interact with heavy metals such as Cr (VI) in the existence of sodium dodecyl benzene sulfonate from Pentachlorophenol (PCPs) [144,145,146,147]. The adsorption of Cr (VI) were inhibited at a pH > 6, while it increases at a pH < 6 due to adsorption sites that available on PE MPs in increasing competition with sodium dodecyl benzene sulfonate. The four types of MPs [low-density polyethylene (LDPE), HDPE, polyvinyl chloride (PVC), and PE] were investigated using three types of heavy metals (Cd^2+^, Pb^2+^, and Cu^2+^). Plastic adsorbed on metal were found in the order of PE > PVC > HDPE > LDPE while metal adsorbed on MPs was Pb^2+^ > Cu^2+^ > Cd^2+^ [132]. The HDPE adsorption of Cd was increased with the increase of pH; in contrast, the efficiency reduced with the increase in salinity. Desorption showed a high tendency towards the adsorbed Cd [148] and posed a greater threat to the biotic environment [149].

### 3.2. Behaviour in Rhizospheric Soil

MPs and NPs adsorb contaminants in agricultural soil and may reach the rhizosphere zone. Different biochemical processes take place in rhizomes around the roots of plants [150]. Plants exude numerous substances such as exudates in the rhizosphere, which change the local environment of plants. Root exudates have been reported to contain phenolic mucilage, various amino acids, sugars, and ectoenzymes. Root exudates play a vital role in the plant rhizosphere to improve nutrient status as root exudates enhance soil structure and affect soil cation exchange capacity, pH, mineral degradation, microbial community, and sorption properties [151,152]. Abiotic stress caused by contaminants changes the root exudates. Vranova et al. [153] reported that under environmental stress, the quality of root exudates can increase 1000-fold from normal values. Abbasi et al. [151] reported that Pb, Cd, and Zn adsorbed on the surface of polyethylene terephthalate (PET) MPs in the wheat rhizosphere zone; the adsorption of Pb, Cd, and Zn in rhizosphere soil is associated with MPs and is available for a longer time to plants. Another study reported that in rice rhizosphere soil, PS MPs and polytetrafluorethylene (PTFE) particles combined with arsenic affect soil properties, available nutrients, soil enzymes, and microorganisms. PS MPs and PTFE combined with arsenic reduced the soil pH, bioavailable arsenic, available nitrogen, and phosphorus in the soil rhizosphere. Only arsenic increased soil organic matter while PS MPs and PTFE reduced soil organic contents. In addition, it also affected soil enzymes such as acid phosphatase, dehydrogenase, soil urease, protease, and peroxidase activity. PS MPs and PTFE and arsenic increased the abundance of Acidobacteria and Chloroflexi while reducing the abundance of Proteobacteria [119]. In the rhizosphere, the abundance of arbuscules, hyphae, and arbuscular mycorrhizal fungi was significantly increased with PS microfibers; this may depend on the alteration in soil structure and water dynamics by PS microfibers [154]. In contrast, PLA microplastic has significant adverse effects on arbuscular mycorrhizal fungi diversity and community structure, possibly due to toxicity associated with the biodegradation of PLA [155]. However, there is an urgent need to conduct research on the effect of MPs and NPs on soil properties, soil enzymes, soil microorganisms, and interaction with other contaminants in the plant rhizosphere.

### 3.3. Interaction with Soil Microbes

Soil microbes are key players in the biogeochemical cycling of elements and for the production of food. Better understanding the response of MPs to soil microorganisms will allow us to better predict the possible consequences as a result of MP pollution. MPs can act as a new host for microorganisms living in soil–plastic interfaces, which could lead to the formation of unique microbial communities [156].

Soil MPs and NPs change the diversity of bacterial and fungal communities. Some recent studies have reported that several types of MPs encourage and inhibit the bacterial community, and enzymatic activity such as Bacteroidetes and Actinobacteria increase their community on the surface of PE MPs [25,157,158,159,160,161]. Polyacrylic and polyester fibers decrease the metabolic activity of microbes [162]. In wheat soil systems, PVC and PE NPs shift microbial communities from Gram-positive to Gram-negative and also decrease xylosidase and β-glucosidase activity by 16–43% [163]. PVC increases Desulfobulbaceae and Desulfobacteraceae while decreasing Sedimenticolaceae and Chromatiaceae due to some antimicrobials which may be attributed to plastic additives [164]. In addition, PS microbeads inhibit Bacteroidetes, Proteobacteria, and Firmicutes due to the possible interaction with reduced soil nutrients as also shown in arsenic-polluted paddy soils [165]. MPs and NPs change soil properties and bacterial communities. Rhizobia can potentially change with changes in the soil matrix [166]. The ingestion of MPs by soil organisms could influence bacterial diversity; e.g., Zhu et al. reported that in the collembolan gut, MPs potentially enhanced bacterial diversity, possibly due to a move in feeding after MP exposure [102]. Some researchers have reported that some plastic microfilms such as PLA, PCL, PHA, PBAT, and starch-based biopolymers types have been approved as fixed C sources to enhance the concentration of fungal species, e.g., Fusarium, Aspergillus, and Penicillium [167,168,169]. Different sizes of plastic have different effects on microbes due to their change in surface area [78]. NPs (<0.1 μm) especially could enter the cell membrane and cause cytotoxic effects [170], due to the bioaccumulation in the cell of filamentous and yeast fungi [15,171,172]. The ability of NPs to enter and accumulate in soil organic debris causes biological effects on microbes, while NPs may be less important for altered soil properties. In addition, water-stable aggregates are important for microbial activity as PS fibers decrease water-stable aggregates, resulting in significant impacts on plant soil health. However, there is no data available on the effects of MPs and fungi on soil–plant systems [173]. Table 1 shows the impact of different types of plastics on soil microorganisms.

An increase in the abundance of MPs and NPs in soil can alter microbial communities, increasing the proportion of microbial communities selected by MPs and NPs. Hence, in combination with other natural substances and in association with the plastisphere, newly introduced MPs affect environmental ecological functions. According to the above discussion, the plastic and microbe interaction may significantly affect the soil–plant interaction, fauna development, and nutrient recycling. Otherwise, it is recommended that the surface of MP be a hotspot for microbial development. Therefore, to better understand the response and function of soil microorganisms, it is necessary to understand MP and NP environmental behaviors. However, understanding of the mechanism of interaction between the microbial community and MPs is still unknown and is an important scientific gap that must be filled to better assess the environmental impact of MPs on soil.

## 4. Fate and Uptake in Agricultural Plants

### 4.1. Transport of Plastic in Root Tissue

**Figure 3:**
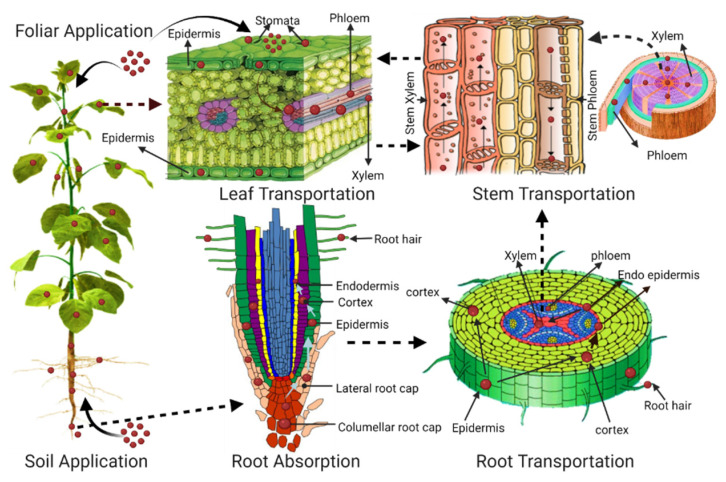
Plant uptake mechanism of plastic by soil application via root absorption and transport pathways from root to stem and stem transport to leaf and fruits. Foliar application reveals the entry of plastic to leaf stomata and later transfer to other parts of the plant. Cure arrow indicates the availability of plastic to plant and the dashed arrow indicates transportation within the plant.

The uptake of MPs and NPs in plants has been detected and plastic particles are especially absorbed on root hairs. Plastic particles have short-term and transitory effects on germination rates and root development as shown in Figure 3 [49]. Given our preliminary knowledge regarding the effects of MPs and NPs in plant systems, it is important to further explore the mechanisms related to MP and NP uptake and accumulation in plant systems, as shown in Table 2 [175].

MPs cannot enter into plant tissues directly as is expected because their large size particles prevent them from entering into the plant cell walls [121]. While NPs can directly enter into the plant cell walls, according to a study of tobacco (*Nicotiana tabacum*), plant cells show that tobacco plants did not uptake 100-nanometer nano polystyrene beads, but 20-nanometer to 40-nanometer beads were taken up [176]. Li et al. [41] reported on treated roots with 0.2-micrometer PS microbeads with labeled fluorescence and it was seen that the 0.2 μm PS fluorescent microbeads were trapped in the cells outside the root cap mucilage usually obvious to the naked eye [41]. In plants, mucilage and exudates act as the first layer of protection, are negatively charged, and have been reported to inhibit positively charged metal uptake of NPs at the outer side of the cell wall [177]. The 0.2-micrometer PS luminescence signals were located on the cell walls of the cortical tissue of the roots and mainly observed in the vascular system, which showed that the beads passed through the intercellular channels, the apoplastic transport system [41]. PS beads were seen along the entire lateral root cap and inside the apical meristem of wheat (*Triticum aestivum*) and lettuce (*Lactuca sativa*) roots. They suggest that these particles in the root cap mucilage stimulate their perception of the cell wall, which is highly insecure due to active cell division, and permit diffusion through the apical meristem tissue. PS beads can only enter the apical meristem through the epidermal layers of whole apical root sections, while casparian is not fully mature. PS luminescence signals were mainly located in the vascular system of the wheat root through confocal images. After 2 h, these signals were observed in the epidermis and xylem vessels and were also visible in the wheat cortical tissue after 12 h. While in lettuce (*Lactuca sativa*) plants, they appear to be confined to vascular tissues. PS beads penetrated to the cortical through gaps in the epidermal cells, while not entering the endoepidermis because the casparian band was permanent. In the lateral root apex, strong PS luminescence signals were deleted in cracks where these particles entered into the cortex and endoepidermis, which showed that small PS bead crack entries were major sites into the lettuce (*Lactuca sativa*) and wheat (*Triticum aestivum*) root xylem [41]. Through a scanning electron microscope (SEM) observation, PS beads were detected in the stele on the secondary roots after 12 h, and after 48 h PS beads were detected in the epidermis and vascular tissue of wheat [41].

Transmission electron microscopy [152] analysis of PS NPs treated with *Allium cepa* root with different concentrations and sizes was previously reported. The cytoplasm of treated cells with NPs was often filled with electron-dense bodies; it is possible that lipid bodies have not yet been mobilized while 1 g L^−1^ are more abundant than 0.1 g L^−1^ NPs PS-treated roots. In these bodies, the endoplasmic reticulum, mitochondria, vacuoles, and dictyosomes were investigated, but PS NPs were detected in both the cytoplasm and vacuoles. They had a cylindrical shape, ranging in size from 25 nm to 130 nm and often appearing in aggregates of 2–5 particles. 25-nanometre PS NPs were observed in the nucleus [44]. The existence of PS NPs in the nucleus suggests that perhaps small PS NPs may also cross the nuclear membrane and inhibit the function and structure of chromatin [178]. However, no study so far demonstrated the uptake and transportation of MPs in the whole plant. Detailed studies are required to understand the mechanisms of uptake and transportation of MPs and NPs in plants.

### 4.2. Translocation of Plastic from Root to Leaves

Plants can uptake or adsorb MPs and NPs in the form of aggregates [179]. Transpiration pull plays a major role in the plant uptake and translocation of plastic particles. According to Li et al.’s [41] findings, plastic particles enter the epidermal tissue of wheat’s primary and secondary roots and are stimulated through the pericycle and moved into the xylem. Inside the central cylinder, these particles, through the xylem, can move to the aerial part of the plant, as shown in Figure 3. Plastic particles were transferred from root to shoot by the vascular system via the transpiration stream. Confocal images confirmed that plastic luminescence signals were traced mainly in the vascular system of the stem. MPs and NPs can travel in microscopic extracellular channels and reach the vasculature accountable for water transportation [43]. Water transportation system supporting NPs can quickly transfer to the stem, leaves, and possibly fruits. Recently, Lian et al. [54] revealed plastic NP uptake and translocation in wheat crops. Confocal microscopy with 3D laser scanning clearly showed that root tips take up PS NPs while for PS NPs in leaves, the fluorescent signal was shielded by the auto fluorescence of plant tissues. Similarly, PS NPs that absorbed in root and shoot xylem were also detected by SEM. PS NPs (100 nm) could be transmitted from root to shoot via the xylem pathway, which may possibly be transported into the grain. Bosker et al. [49] found clusters of plastic particles in the leaf and epidermis of *Lepidium sativum* through epifluorescence and confocal microscopy after 48 and 72 h of exposure with MPs. The PS beads (0.2) were observed by the SEM in the wheat’s outer side of the cell walls in the stem xylem and also detected in the intercellular space of lettuce leaf veins. Li et al. [42] confirmed that PS NPs and MPs can accumulate in the stem and leaves tissue of the cucumber plant; 500-nanometer and 700-nanometer MP particles were observed in the first fruit interspace tissue of a calyx cross-section. They also suggested, based on the results, that PS NPs can accumulate in the flowers and fruits of cucumber plants.

Another possible pathway for plastic entry into the plant leaf is through the stomata via foliar application [180]. Sun et al. [46] revealed that stomatal uptake is one possible pathway for NPs into the leaves and then moves to the vasculature. Micro-fluorescent PS NPs had a high rate in the stems and a low rate in the root of the maize plant, and fluorescence microscope imaging analysis also indicates a high occurrence of PS NPs in and around the vascular system of the stem. These results show NP movement from the leaves to the stems and later from the stems to the roots through the vascular bundle. Similarly, another study confirmed that PS NPs attach to the plant’s stomata, penetrate through the phloem and reach the roots of the lettuce plant [47]. The uptake and accumulation of MPs and NPs in the plant shoot are currently still very limited. Detailed studies are required on the mechanisms of the uptake and transportation of MPs and NPs in plant shoots.

## 5. Effects of MPs and NPs on Plants

### 5.1. Effects of MPs and NPs on Plant Physiology

**Figure 4:**
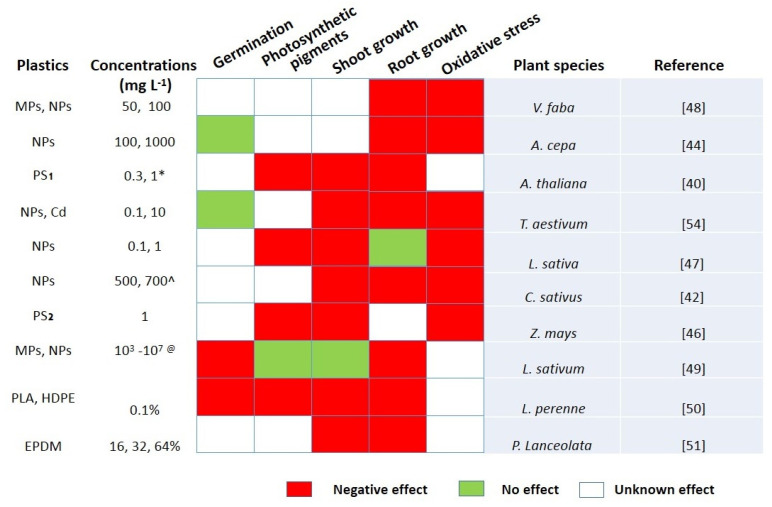
Effects of MPs and NPs on plant Physiology and biochemical indicators. PS_1_ represents PS (SO_3_ and NH_2_), PS_2_ represents (COOH and NH_2_), * represents g Kg^−1^, ^ represents nm, and ^@^ represents particles L^−1^.

The agroecosystem appears to be highly contaminated by both plastics, which negatively affects plant growth and development. Although evidence on the impact of MPs and NPs on agricultural plants is currently scarce [181], recently, a few studies reported the effects of MPs and NPs on vascular plants. Plant stress responses to MPs and NPs particles have mainly focused on physiological and biochemical indicators as shown in Figure 4.

In the last five years, only four studies have reported on the impact of MPs and NPs on seed germination. For example, in garden cress (*Lepidium sativum*), germination rates were reduced by 56%, 46%, and 21% after 8 h at 10^3^ to 10^7^ particles m L^−1^ of NPs and MPs (50, 500, and 4800 nm), respectively [49]. The reduction in germination was due to plastic particles blocking pores or spore surfaces, reducing water uptake [182]. Similarly, perennial ryegrass (*Lolium perenne*) germination rates were reduced by 6% and 7% after 30 days of exposure to PLA (0.1% *w*/*w*) and (10 mg kg^−1^) MP fibers, respectively [50]. In contrast, wheat (*Triticum aestivum*) germination rates have no effect at 10 mg L^−1^ of PS NPs (100 nm) [54]. Furthermore, in onion (*Allium cepa*), 0.01, 0.1 and 1 g L^−1^ of PS NPs (50 nm) have negligible effects on germination rates after 72 h [44]. Overall, short-term studies suggest that MPs and NPs have no significant effects on germination indicators.

Additionally, the effects of MPs and NPs on plant growth indicators in the agroecosystem have been investigated through seven studies. For example, *Allium cepa* root growth was reduced by 41.5% at 1 g L^−1^ compared to control treatments after 72 h of PS NPs (50 nm) [44]. Similarly, 10–100 mg L^−1^ irregular-shaped PE beads decreased the root length of duckweed (*Lemna minor*) over seven days of exposure [53,183]. De Souza et al. [154] elucidate that six different MPs (polyester fibers, polyamide beads, and fragments of polyethylene, polyester terephthalate, polypropylene, and polystyrene) at 2% concentration in the soil had an impact on the performance of spring onion (*Allium fistulosum*). PES and PS MPs had a significant increase of >97.5% on root biomass while a lesser effect was shown for PEHD, PP, and PET PP, which decreased (~>75%) the average root diameter. The dry biomass of onion bulbs was significantly decreased (probability > 75%) in polyamide bead-treated plants, while all MPs were significantly altered from the control concerning the dry weight of onion bulbs. Moreover, the total root length significantly increased under all analyzed MPs (~>75%) and the total average root diameter decreased (~>75%) as well. With a significantly increased biomass of the root (longer and finer), the total area of the root was also increased with all MPs (~>75.0%). PA decreased root tissue density; PS and PES stimulated the enhancement of such responses and non-significant impacts were observed for PET, PEHD, and PP [154]. Arabidopsis thaliana fresh weight was reduced by a 50% average at all concentrations of NPs (PS-SO_3_H and PS-NH_2_), while greater levels of inhibition were observed at higher concentrations. A significant reduction in plant length was reported only with NPs (PS-NH_2_) up to 15% and root length was reduced by a 30% average at all concentrations of NPs (PS-SO_3_H and PS-NH_2_) while greater effects were observed at higher concentrations [40]. Another study observed PS plastic (5 μm and 100 nm) with concentrations of 10, 50, and 100 mg L^−1^ for 48 h in broad beans (*Vicia faba*). PS plastic (5 µm) significantly decreased root elongation by approximately 60% at 50 mg L^−1^ and 90% at 100 mg L^−1^ while root length decreased by approximately 50% at 50 mg L^−1^ and 60% at 100 mg L^−1^. In addition, PS plastic (5 µm) significantly decreased fresh and dry weight at all exposure concentrations, while PS plastic (100 nm) remained nontoxic at 10 and 50 mg L^−1^ while 100 mg L^−1^ concentration showed a significant effect as compared to the control [48]. In cucumber (*Cucumis sativus*) plants, root lengths were reduced by approximately 12% at 100 nm and by approximately 8% at 300 nm of exposure of PS NPs, while no effects were observed at 500 nm and 700 nm. Root diameter declined at 100 nm, whereas 300-nanometer, 500-nanometer, and 700-nanometer treatments showed no effects compared to the control [42]. Recently, another study reported the combined effects of Cd (0.1 and 10 mg L^−1^) with PS beads in wheat (*Triticum aestivum*) crops for 21 days. Co-exposure treatment decreased the dry biomass of root and shoot [54] by 14.9%. In maize (*Zea mays*), two, five, and seven days’ exposure of foliar application of PS NPs (PS-NH_2_ and PS-COOH), positively charged NPs PS (PS-NH_2_) particles with a concentration of 1 mg L^−1^, had inhibitory effects on maize growth. Negatively charged PS NP (PS-COOH) particles with a concentration of 1 mg L^−1^ reduced the fresh leaf weight of seedlings by 8% as compared to the control [46]. It can be predicted that PS charge also plays a major role and differently charged particles cause different effects on plant growth. However, more detailed investigations are needed in agricultural soil to understand the risk of this mounting pollution on the food chain.

MPs and NPs have both positive and negative concentration-dependent effects on plant photosynthetic indicators and chlorophyll contents. For example, through foliar application PS NPs (PS-NH_2_ and PS-COOH) on maize (*Zea mays*) at different days (two, five, and seven), PS-NH_2_ had significant effects (24–27%) on plant photosynthetic activities in maize leaves as compared to PS-COOH after a seven-day exposure [46]. Similar results were reported in lettuce (*Lactuca sativa*) after exposure to 23-nanometer PE MPs for 28 days [184]. Arabidopsis thaliana were exposed to PS NPs (PS-SO_3_H and PS-NH_2_) with concentrations of 0.3 and 1 g kg^−1^. The results showed that PS-NH_2_ (1 g kg^−1^) significantly inhibited chlorophyll contents of Thale cress (*Arabidopsis thaliana*) while PS-NH_2_ (0.3 g kg^−1^), PS-SO_3_H (0.3 and 1 g kg^−1^), and the control remained the same [40]. However, these concentrations are unrealistic for the actual environment. In contrast, a recent study highlighted the lack of effects of PS NPs (50 nm and 500 nm) (at 10^2^ and 10^6^ particles mL^−1^) on photosynthesis efficiency and chlorophyll in duckweed (*Spirodela polyrhiza*) after 120 h [185]. The same was seen with the high concentration of MP (PE) (1–1000 μm) exposure to duckweed (*Lemna minor*) for seven days [53,179]. It’s hard to reach any conclusion due to the contradictory reports on the effects of MPs and NPs on physiological indicators. We suggest that further detailed studies are required to understand the impact of MPs and NPs on chlorophyll and photosynthetic efficiency.

### 5.2. Effects of MPs and NPs on Plant Biochemical Indicators

Reactive oxygen species are produced as a normal product involved in plant cell metabolism. Antioxidant systems are responsible for the function of SOD, POD, and CAT to reduce the damage caused by environmental stress by reducing the number of reactive oxygen species and free radicals [186,187,188]. ROS are formed at the cellular and various organelles locations such as cell walls, endoplasmic reticulum, plasma membranes, apoplasts, peroxisomes, mitochondria, and chloroplasts [189,190].

Interestingly, several recent reports provide evidence that MPs and NPs can induce oxidative damage to plants. For example, in maize (*Zea mays*), PS-NH_2_ have significant effects on the antioxidant system compared to PS-COOH over seven days of exposure. PS-NH_2_ and PS-COOH enhance SOD, POD, and CAT activity by 200%, 110%, and 192% versus 99.2%, 63.4%, and 33.4%, respectively [46]. In broad beans (*Vicia faba*) with exposed PS plastic (5 μm and 100 nm) with concentrations of 10, 50, and 100 mg L^−1^, PS plastic (5 µm) decreased CAT enzyme activity by ~15%, 65%, and 87% at 10, 50, and 100 mg L^−1^ concentrations, respectively, and PS plastic (100 nm) increased CAT enzyme activity by ~78%, 81%, and 12% at 10, 50, and 100 mg L^−1^ concentrations, respectively. PS plastic (5 µm) increased SOD enzyme activity by ~59%, 131%, and 222% at 10, 50, and 100 mg L^−1^ concentrations, respectively, and PS plastic (100 nm) increased CAT enzyme activity by ~13%, 172% and 59% at 10, 50, and 100 mg L^−1^ concentrations, respectively, as compared to the control. [48]. In lettuce leaves, foliar application of PS NPs at 0.1 and 1 mg L^−1^ concentrations increased electrolyte leakage by 18.6% and 25.5%, respectively, and total antioxidant contents and soluble proteins were significantly reduced by 26% and 17.9%, respectively, at 1 mg L^−1^ as compared to the control. [47]. Another study reported that PS NPs combined with Cd substantially decreased SOD activity in wheat leaves and roots and POD and CAT activity remained the same [54].

In wheat (*Triticum aestivum*) treated with Cd and PS NPs effect MDA contents, Cd alone and combined with PS NPs significantly increased MDA content by 58–61% in roots and 35–38% in leaves and PS NPs alone reduced MDA content in leaves [54]. In *Vicia faba*, PS plastic (100 nm) decreased MDA enzyme activity by ~39% with concentrations of 50 mg L^−1^ and increased MDA enzyme activity by ~37% with concentrations of 100 mg L^−1^ [48]. PS-NH_2_ and PS-COOH had no effects on MDA activity after two days of exposure to a maize leaves. After seven days of exposure, PS-NH_2_ significantly increased MDA contents by 176% and PS-COOH induced no significant changes [46]. NPs block cell wall pores in the leaf vessels, which disturbs the transportation of nutrients. Due to NPs in the leaf vessels, the content of MDA continued to be increased to a certain degree, which caused maximum oxidative damage to the maize leaves [46,191,192].

According to these studies, NPs have significant effects on plant biochemical enzymes, but different plastics (MPs and NPs) have different effects on each plant species. To better understand, more studies are needed on the impacts of different types of plastic (MPs and NPs) on plant interaction, translocation, and the possible effects of soil and foliar applied plastics, which might help for risk assessments of plastic and food safety.

## 6. Conclusions and Future Prospective

Plastic contamination is a rapidly increasing concern throughout the world and has been recently listed as the second most emerging environmental and ecological issue after global warming. Justifiable concern has been expressed in the published literature from world environment organizations concerning the possible presence of both plastics in terrestrial environments and their consequent effects on human health. Mainly, this monograph explores the potential risks of plastic from intensive modern agriculture in the soil system and their fate and transport in agricultural plants.

Finally, it establishes that MPs and NPs, despite having some soil remediation potential, pose harmful effects on plants growth and soil ecology. Occurrences of REEs in surface water and groundwater are caused by three main factors: the weathering of deposits, leachate of mining areas, and discharges of industrial waste. Mulching, organic fertilizer, and effluents of wastewater treatment plants were the primary sources of both plastics in agricultural soil. Agricultural practices such as irrigation, tillage, and fertilization enhance the transport of both plastics in subsurface soil. Soil organisms, including earthworms, mites, and collembolans, induce transportation through casting, ingestion, egestion, and adhesion to their exterior skin.

The adsorption capacity of plastic, which affects the availability of nutrients to the plant as well as adsorbs toxic contaminants, might have negative effects on soil microbial communities. The adsorbed contaminants may be available for a longer time in the soil and affect the growth of the plants.

NPs can directly enter into the plant tissue because of their small size. Plants can uptake NPs directly from the growth media, then be transported to the aerial parts of the plant through the xylem. Foliar exposure of plastic enters the stomata and might be translocated to the plant root through vascular bundles. Additionally, both plastics have toxic effects on physiological parameters and enzymatic activity of agricultural plants. Finally, we make the following specific points regarding future research in the environment, according to the current literature review:

- More clarifications are required for the quantification of decomposition and understanding the mechanisms behind the degradation rate in agricultural soil.
- Long-term batch studies are required to understand the sorption/bioavailability potential of plastic in soil environments.
- More data are required for a better understanding of plastic behavior in different soils types and their interaction with soil microorganisms, especially in rhizosphere soil.
- Root exudates and mucilage are major barriers to contaminant entry in the plants; detailed studies are required for a better understanding of how plastic deals with entry into the plants.
- Soils are probably the major sink for NPs. However, the fate and behavior of NPs in soil environments remains poorly understood. Additionally, full-lifecycle studies on the interactions between NPs and plants are quite scarce. There is an urgent need to explore the mechanisms behind the toxicity of NPs in plants.
- The current knowledge about the bioaccumulation of NPs in plants and understandings of dietary uptake are limited, even though less information about their fate and behavior are inside the food web. Critically the main question in this context is if and how the uptake, bioaccumulation, and trophic transfer of NPs differ respectively to other pollutants.
- Interactions of NPs with other engineered nanoparticles under different growth media have not been explored yet. Thus, more conclusive data is required for a better understanding of NPs in agriculture systems to overcome this emerging issue.

## Figures and Tables

**Table 1:** The impact of plastics on soil microorganisms.

Plastics	Level (%)	Effects on Microorganism	References
PE	1, 5, 10, 20	Decreased xylosidase and β-glucosidase activity by 16–43% and MPs increased the soil microbial biomass (+43.6%).	[163]
PVC	1, 5	Shown positive effects on acidobacteria, bacteriodietes, and hydrolase and urease enzymes while negative effects are shown on Sphingomonadaceae and the Fluorescein diacetate enzyme.	[25,174]
PE	NA	MPs provided habitat to actinobacteria, bacteroidetes, Proteobacteria, gemmatimonadetes and Acidobacteria. Additionally, colonies of bacteria significantly varied in structure from those in the surrounding soil.	[157]
PE	5	In the fertilized soil, MPs significantly enhanced the bacterial and fungal community. MPs seem to indicate the selective impact on microbes and cause a serious hazard to biogeochemical cycles and microbes ecology.	[158]
PE	0.076 g kg^−1^	Increased Bacteriodietes, Acidobacteria, Nitrospirae, Gemmatimonadetes and diminished effect on nutrient cycling as well as positive effect on catalase urease enzymes.	[25]
PVC	0.1	Gut bacterial diversity increased and negative impact on soil macro- and micro-organisms.	[102]
PS	0.2, 0.4, 0.8	Positive and negative effects of numerous Pro Firmicutes, teobacteria, and Bacteroidetes in various MP concentrations.	[165]
PVC	NA	PVC increases Desulfobulbaceae, and Desulfobacteraceae and decreases Sedimenticolaceae and Chromatiaceae.	[164]
PE, PLA	0.1, 1 and 10	MPs change the AMF diversity and structure that depend on their concentration level and type. Enriched with Ambispora (10% of PLA and PE), Archaeosporaceae (PLA 10%), and PLA have a negative impact on plant physiology i-e fresh/dry Biomass and Chlorophyll content.	[155]

**Table 2:** Accumulation of plastics in plants under different exposure conditions.

Plants	Plastic Types	Time (Days)	Media	Accumulation	Reference
*Z. mays*	PS (NH_2_, COOH)	28	Soil	Both types of plastic accumulate in leaves and further transport to the roots.	[46]
*L. sativa*	PS NPs (0.1 and 1 mg L^−1^)	35	Soil	NP uptake by plant leaves by stomata and translocation downwards to the plant.	[47]
*C. sativus*	PS (100, 300, 500, 700 nm)	65	Hydroponics	PS uptake by the root and future transport to the leaves, flower, and fruit through the stem.	[42]
*A. cepa*	PS (0.01, 0.1, 1 g L^−1^)	72 h	Hydroponics	PS enters different cellular compartments.	[44]
*A. thaliana*	PS (SO_3_H, NH_2_)	35	Soil	Positive-charge NPs have a great effect on the root and their uptake and internalization as compared to negative-charge particles.	[40]
*T. aestivum, L. sativa*	NPs 0.2 and 2 μm	20 10	Sandysoil, hydroponics	PS enters the root through crack-entry mode and through transpiration, which pulls PS transport from root to shoot.	[41]
*L. sativum*	MPs and NPs (10^3^–10^7^ P mL^−1^)	72 h	Filter paper	Microplastic accumulates in the pores of the L. sativum seed and delays germination.	[49]
